# Highly effective fat suppression in clinical T1-weighted imaging of ischemic and non-ischemic heart disease with DeSPAIR

**DOI:** 10.1186/1532-429X-14-S1-O53

**Published:** 2012-02-01

**Authors:** Wolfgang G Rehwald, Christoph J Jensen, Elizabeth Jenista, Stephen Darty, Deneen Spatz, Raymond J Kim

**Affiliations:** 1 Siemens Healthcare, Chapel Hill, NC, USA; 2 Duke Cardiovascular MR Center, Duke University Medical Center, Durham, NC, USA

## Summary

Complete fat suppression is highly desirable for inversion recovery (IR) imaging of both ischemic and non-ischemic heart disease. It allows for improved visualization of scar without confounding fat signal, and for assessment of fatty infiltrations, which both appear bright on T1-weighted contrast-enhanced images. For example, arrhythmogenic right ventricular dysplasia (ARVD) is characterized by fibrofatty replacement of the right ventricular (RV) myocardium. Screening tests are critical as patients may be asymptomatic, but at increased risk for sudden cardiac death. Such tests must differentiate myocardial fibrosis from fatty infiltrations, as fatty infiltrations without RV fibrosis have been observed in healthy subjects. Standard fat saturation works poorly in clinical protocols, prompting us to design a double Spectral Selection Attenuated Inversion Recovery (double SPAIR, DeSPAIR) module that completely nulls fat signal in IR sequences. In 22 patients with ischemic and non-ischemic heart disease, we demonstrate excellent performance of this technique compared to standard fat saturation.

## Background

Protocols that impart T1-weighting by an IR pulse, such as delayed enhancement, can incorporate a standard fat saturation pulse to suppress fat, but perform poorly with clinically useful readout lengths. Alternatively, sequences without an IR pulse can null fat by a fat-selective inversion using SPAIR. Unfortunately, this approach cannot be readily applied to sequences with an IR for T1-weighting, since fat would experience the IR pulse and would not be fully recovered when the SPAIR pulse is applied, resulting in such a short effective inversion time (TI_fat) that fat nulling is impossible. We developed a method to overcome this problem by combining a double SPAIR (DeSPAIR) module with an IR pulse to suppress fat while maintaining T1-weighting.

## Methods

**Figure 1:**
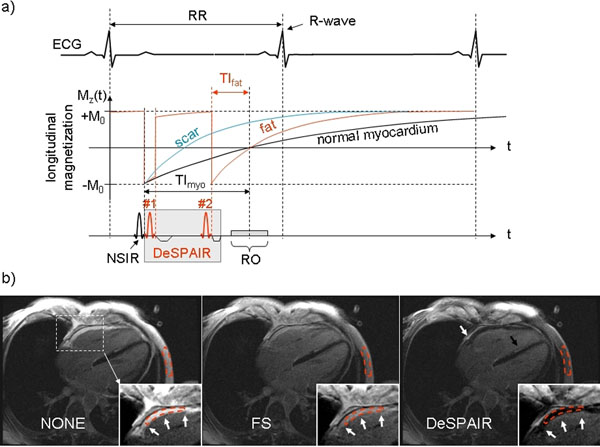
a) shows the DeSPAIR module integrated into a segmented IR gradient echo sequence and the resulting T1 recovery curves. NSIR and SPAIR pulses are timed to null myocardium and fat at the k-space center. Typical parameters at 1.5T (MAGNETOM Avanto, Siemens Healthcare) and 3T (Verio) were, respectively: TI adjusted to null normal myocardium (300-400 ms), trigger pulse 2, fov 360 x 270 mm, matrix 256 x 125, segments 21, flip angle 25° (15°), receiver bandwidth 130 (399) Hz/pixel, TE 3.85 (1.66) ms, TR 8.9 (4.4) ms, slice thickness 7 (6) mm. 1b) shows delayed enhancement images at 3T in an ARVD patient without fat suppression, FS, and DeSPAIR. Fat suppression was poor by FS, but excellent by DeSPAIR. Note the fatty infiltration of the RV freewall (3 white arrows), pericardial effusion (white arrow) only seen in the DeSPAIR image, and septal fibrosis (black arrow).

The DeSPAIR module integrated into an IR sequence is shown in figure 1a. SPAIR #1 immediately follows the non-selective IR (NSIR) pulse to re-invert fat magnetization and keep it at +M0. To null fat, SPAIR #2 follows at time TI_fat prior to readout (RO) of the k-space center. Normal myocardium is nulled by the NSIR placed TI_myo prior to the k-space center. Dark myocardium, dark fat and bright scar result in the image. We acquired 3 images per patient in 22 patients (9 at 1.5T, 13 at 3T, 4 ischemic, 18 non-ischemic) using no fat suppression (NONE), fat saturation (FS), and DeSPAIR. In each image, we measured signal-to-noise (SNR) in multiple red dashed fat regions of interest (ROI) (figure 1b). To quantify suppression efficiency, SNR of FS and DeSPAIR in each ROI was normalized to SNR of NONE in the same ROI and expressed as relative SNR in percent. An ANOVA with Bonferroni correction was applied to test for statistical differences between groups NONE, FS, and DeSPAIR. Cavity and normal myocardium SNR were measured to evaluate if FS or SPAIR would affect SNR.

## Results

Typical delayed enhancement images in an ARVD patient using NONE, FS, and DeSPAIR at 3T are shown in figure 1b. Visual inspection showed excellent suppression of fatty infiltration in the RV by DeSPAIR (3 white arrows), and hardly any by FS. Septal hyperenhancement was present with and without fat suppression, indicating fibrosis (black arrow). Statistical analysis of relative SNR revealed significantly suppressed fat by DeSPAIR at 1.5T and 3T (see table 1). Fat was not suppressed by FS at 1.5T and significantly suppressed at 3T, but less than by DeSPAIR (table 1). Neither cavity nor myocardial SNR were statistically different (p>0.05) between any of the techniques, at both field strengths.

**Table 1:** Relative Fat SNR, Cavity and Myocardial SNR

field strength	1.5T			3T		
sup-pression method	NONE	FS	DeSPAIR	NONE	FS	DeSPAIR

relative fat SNR	100%	101.63% ± 29.92% #	39.52% ± 19.87% *	100% *	68.79% ± 28.88% *	32.72% ± 15.53% *

cavity SNR	19.75 ± 10.29	25.13 ± 12.41 #	16.91 ± 9.28 #	49.82 ± 37.16	49.26 ± 32.29 #	44.92 ± 32.95 #

myocardial SNR	3.78 ± 2.40	5.59 ± 3.28 #	3.85 ± 2.05 #	7.46 ± 5.36	6.79 ± 4.44 #	6.49 ± 5.34 #

shown: mean ± stdev *: statistically different from NONE (p < 0.001) #: statistically identical to NONE (p > 0.05).

## Conclusions

DeSPAIR reliably nulled fat signal at both field strengths for clinically relevant RO lengths. DeSPAIR performed much better than FS at both field strengths, but the difference was particularly striking at 1.5T. DeSPAIR required no manual parameter adjustment and no post processing — only a simple change to an existing sequence. In clinical practice, DeSPAIR can help establish the diagnosis of ARVD even in early stages, and potentially improve patient outcome.

## Funding

NIH grant 5ROIHL064726-07.

